# A novel rhamnolipid-producing *Pseudomonas aeruginosa* ZS1 isolate derived from petroleum sludge suitable for bioremediation

**DOI:** 10.1186/s13568-017-0418-x

**Published:** 2017-06-08

**Authors:** Tao Cheng, Jibei Liang, Jing He, Xingcui Hu, Zhiwei Ge, Jianhua Liu

**Affiliations:** 1 Marine Biology, Ocean College, Zhejiang University, 1 Zheda Road, Dinghai District, Zhoushan Campus, Marine Science Building, Room 379, Zhoushan, 316000 Zhejiang China; 2 Marine Functional Compounds, Ocean Research Centre of Zhoushan, Zhejiang University, Zhoushan, 316021 Zhejiang China; 3 Analysis Center of Agrobiology and Environmental Sciences, Zhejiang University, Hangzhou, 310058 Zhejiang China

**Keywords:** Bioremediation, Biosurfactant, Emulsification, Oil sludge, Rhamnolipids, *Pseudomonas aeruginosa*

## Abstract

Petroleum pollutants emulsified by biosurfactants facilitate uptake and biodegradation by environmental microbes. In this report, we show the characterization of an indigenous surfactant-producing crude-oil-eating microbe isolated from petroleum-sludge in Zhoushan islands, China, where one of the national strategic petroleum reservoirs is located. We examined biosurfactant activities using surface tension analysis on mixed culture originated from oil-sludge microbes. In parallel, dynamics of individual microbial populations in cultures were monitored using the terminal fluorescence labeled (TFL)-RFLP method. Biosurfactant activity was found to be associated with a dominant microbial species designated as ZS1 (Zhou-Shan isolate 1). Cell-free supernatant from the ZS1 culture exhibited 100% emulsification index against crude oil and reduces surface tension to 26.5 mN/m. Sequence-based analysis suggested that the ZS1 belongs to the group of *Pseudomonas aeruginosa*. LC–MS/MS analysis indicated that the rhamnolipids produced by the ZS1 consisted of 7 monorhamnolipid and 11 dirhamnolipid homologues (RL7-11), two of which were novel. Maximum yield of rhamnolipids in shake-flask ZS1 culture could reach 44 g/l. Furthermore, we showed that ZS1 was capable of growing in MS medium supplemented with 1% crude oil as sole carbon source, in which cell mass increase coincided with the formation of crude-oil emulsion. Half of the crude oil was consumed by the ZS1 in 12 days. Taken together, our results show that ZS1 produces high level of rhamnolipids that effectively emulsify crude oil accelerating its uptake and degradation. Hence, it is suitable for bioremediation of petroleum pollutants in Zhoushan coastal areas.

## Introduction

Petroleum leaks and spills are often linked to accidents resulting from activities related to the petrochemical industry involving oil drilling rigs, transportation tankers and pipelines, and storage reservoirs (Holliger et al. 1997). It has been estimated that at least 400,000 metric tons of natural crude oil leaked to the environment every year worldwide (Kvenvolden and Cooper 2003). Chemically synthesized surfactants are routinely used for removal of petroleum pollutants (Kanaly and Harayama 2010). However, most chemically synthesized surfactants are only partially and slowly biodegraded by the environmental microbes, some of which are known to be toxic to animals, ecosystems, and humans, contributing to secondary pollutants (Henkel et al. 2012; Murphy et al. 2005).

Biosurfactants are surface-active amphiphilic compounds produced by living cells and provide a promising alterative to chemically synthesized surfactants, since they are low toxicity, high biodegradability, and ecological safety (Sponza and Gök 2010). A number of microorganisms have shown to produce biosurfactants, for example, *Acinetobacter calcoaceticus*, *Bacillus subtilis*, *Pseudomonas aeruginosa*, and *Starmerella bombicola* produce emulsan, surfactin, rhamnolipids, and sophorolipids, respectively (Cooper et al. 1981; Shoham et al. 1983; Sponza and Gök 2010; Zajic and Supplison 1972). Rhamnolipids are among the most effective surfactants in removing crude petroleum oil from contaminated sites (Desai and Banat 1997; Lang and Wullbrandt 1999; Santa Anna et al. 2002; Soberón-Chávez et al. 2005).

Rhamnolipids are glycolipids whose amphiphilic structure consists of one or two l-rhamnose moieties (rha) linked in a 1,2-glycosidic linkage to up to three β-hydroxy fatty acids with chain lengths ranging from eight to sixteen (Abdel-Mawgoud et al. 2010; Edward and Hayashi 1965). The most abundant species of rhamnolipids produced by *P. aeruginosa* are monorhamnolipids α-l-rhamnopyranosyl-β-hydroxydecanoyl-β-hydroxydecanoate (rha-C_10_-C_10_) and α-l-rhamnopyranosyl-β-hydroxydecanoate (rha-C_10_) and dirhamnolipids α-l-rhamnopyranosyl-α-l-rhamnopyranosyl-β-hydroxydecanoyl-β-hydroxydecanoate (rha-rha-C_10_-C_10_) and α-l-rhamnopyranosyl-α-l-rhamnopyranosyl-β-hydroxydecanoate (rha-rha-C_10_) (Abdel-Mawgoud et al. 2010).

Extra rhamnose ring in dirhamnolipids (e.g., rha-rha-C_10_-C_10_) confers high hydrophilicity compared to monorhamnolipids (e.g., rha-C_10_-C_10_). On the other hand, extra fatty acid chain in dilipidic rhamnolipids (e.g., rha-rha-C_10_-C_10_) exhibits high hydrophobicity compared to monolipidic rhamnolipids (e.g., rha-rha-C_10_). These properties affect the rhamnolipid’s amphiphilicity that determines their stability in aqueous phase, capability to solubilize hydrophobic organic compounds, and bioavailability (Mata-Sandoval et al. 1999). Monolipidic rhamnolipids such as rha-C_10_ and rha-rha-C_10_ displayed high critical micelle concentrations (CMC) values up to 200 mg/l, whereas dirhamnolipids like rha-rha-C_10_-C_10_ and monorhamnolipid rha-C_10_-C_10_ show relatively low CMC values from 40 to 120 mg/l (Abdel-Mawgoud et al. 2009; Haba et al. 2003). Because of a wide range of amphiphilicities, whole mixture of rhamnolipid congeners produced by *P. aeruginosa* can serve as good surface-active agents in some industrial applications in a cost-effective manner (Abalos et al. 2001; Deziel et al. 1999; Ma et al. 2016).

Rhamnolipid biosynthesis is believed to be catalyzed by the rhamnosyltransferase 1 chain A (RhlA) and chain B (RhlB) and rhamnosyltransferase 2 (RhlC). RhlA catalyzes the formation of β-hydroxyalkanoyl-β-hydroxyalkanoic acid (HAA) from β-hydroxyacyl-APC or β-hydroxyacyl-CoA (Rehm et al. 1998). RhlB catalyzes the formation of monorhamnolipid from HAA and dTDP-l-rhamnose (Ochsner et al. 1994a, b). And RhlC catalyzes the formation of dirhamnolipid from monorhamnolipid and dTDP-l-rhamnose (Rahim et al. 2001). Exogenous expression of those genes in *E. coli* permits synthesis of rhamnolipids (Ernst 1997; Meyer 1994).

Many microbes are known to be able to degrade petroleum hydrocarbons in aquatic systems such as oceans (http://www.metamicrobe.com/petroleum-microbiology/oil-bioremediation-bacteria.html). *Alcanivorax borkumensis* is one of the well characterized hydrocarbonoclastic marine bacterium (Akihiro et al. 2003; Yakimov et al. 2007) that produces glucolipids (Naether et al. 2013). *P. aeruginosa* is also a well characterized oil-eating bacterium (Varjani and Upasani 2016) that produces rhamnolipids (Zhang and Miller 1992). Biosurfactants emulsify crude oil facilitating oil uptake and biodegradation by the oil-eating bacteria.

In this report, we describe a terminal fluorescence labeled (TFL)-RFLP-mediated screening method, from which we obtained a rhamnolipid-producing *P. aeruginosa* ZS1 isolate originated from petroleum sludge in Zhoushan islands. ZS1 isolate produces a mixture of rhamnolipid homologues whose efficacy and capacity against crude oil under various environmental conditions are higher than that of synthetic surfactant SDS. Ex situ bioremediation analysis of ZS1 isolate in shake-flask containing MS medium supplemented with 1% crude oil as sole carbon source at 30 °C indicates that 50% of the crude oil could be consumed in 12 days after growth. We propose that this indigenous ZS1 isolate is suitable for ex situ and in situ bioremediation of petroleum pollutants in Zhoushan islands where national strategic petroleum reservoirs are located. Besides, it has a great potential for large-scale production rhamnolipids using glycerol as sole carbon source.

## Materials and methods

### Strain isolation and culture manipulation

Petroleum sludge was collected from a loading dock of oil tanks at the Sanjiang Ferry Terminal in Zhoushan islands, Zhejiang Province, China and stored at 8 °C. To cultivate microbes originating from sludge, 0.1 mg sludge was added into a Falcon tube containing 3 ml mineral salt (MS, 1 l contains: 0.6 g Na_2_HPO_4_, 0.2 g KH_2_PO_4_, 4.0 g NaNO_3_, 0.3 g MgSO_4_, 0.01 g CaCl_2_, 0.01 g FeSO_4_) (Zajic and Supplison 1972) medium supplemented with 2% yeast extract (YE) (Thermo Fisher Biochemicals Ltd, Beijing, China) overnight at 30 °C with shaking for 3 days. The resulting culture was used as seed to inoculate the fresh MS medium supplemented with 2% yeast extract, 2% glucose, or 1% crude oil. Cell growth was monitored by either colorimetric (optical density at the wavelength of 600 nm) or gravimetric methodologies (cell dry weight). All growth curve analyses in this study were carried out in triplicate. Standard rhamnolipids containing Rha-C10-C10 and Rha-Rha-C10-C10 were used as references (Cat: BMO156, Lot: 14031-2, BALX Biotech Co. Ltd., Tianjin, China).

### Surface tension analysis

Surface tension in supernatant or solution containing purified RL7-11 was determined by using the BZY-B surface tensiometer (Fangrui Instrument Co. Ltd., Shanghai, China) with the du Nouy ring method. It was operated at the room temperature 25 °C in multiplicate typically between 3 and 5 repeats. Relevant fresh medium was used as control. The instrument was always calibrated using distill water (72 mN/m) and ethanol (22 mN/m) prior to use.

In analysis of CMC, 10% of RL7-11, RL-STD (standard rhamnolipid molecules from BALX Biochem Co. Ltd.) and SDS solutions (w/v) was subjected to twofold serial dilutions. The resulting dilutions were subjected to surface tension analysis. CMC was referred as the minimum concentration required for reducing surface tension to the minimum level. Similarly, in analysis of CMD, serial diluted supernatants were subjected to surface tension analysis. CMD was defined as the maximum dilution required for reducing the surface tension to the minimum level.

### Oil-spreading assay

Oil-spreading assay was applied for estimation of surfactant activity quantitatively following the method by Morikawa et al. (2000) with a minor modification for better preservation of the round shape of the oil-spreading zone (OSZ). Briefly, 50 ml of distilled water was added to a petri dish with a diameter of 15 cm. Subsequently 5 μl of the crude oil was added on to the surface of water, which was quickly spread out to form a thin layer. Then, a drop of 20 μl crude oil was added and formed the round shaped raft on the surface. To test the surfactant activity, 5 μl supernatant was added on to the center of the crude oil raft and waited for 30 s to allow formation of the oil-spreading zone (OSZ). The image of OSZ in petra dish was taken and subjected to NIH ImageJ analysis (http://www.imagej.nih.gov) for the area of OSZ and DISH in Pixels. The area of OSZ was calculated by the following formula:

$${\text{Area}}_{\text{OSZ}}={\text{Area}}_{\text{DISH}}\left( {\text{Pixel}}_{\text{OSZ}}/{\text{Pixel}}_{\text{DISH}} \right)$$

where Area_OSZ_ and Area_DISH_ are the area of OSZ and DISH (respectively); Pixel_OSZ_ and Pixel_DISH_ are the pixels determined by the ImageJ software. A standard curve between quantity of RL7-11 and size of OSZ could be obtained based on this method.

### Orcinol-sulfuric acid assay

A colorimetric orcinol-sulfuric acid assay method (Marchant and Banat 2014) was used to estimate rhamnolipid concentration. Briefly, 1 vol. of supernatant was mixed with 9 vol. of freshly prepared 0.19% orcinol in 53% sulfuric acid. The sample was incubated at 80 °C for 30 min, cooled to RT for 15 min, and subjected to OD measurement at the wavelength of 421 nm. Standard curve between rhamnose and OD_421_ was prepared using rhamnose (Sigma-Aldrich Co. LLC, Shanghai, China) within a range of 0–50 μg/ml. Based on the standard curve, rhamnolipid concentration was estimated as 3 times of the rhamnose concentration according to the OD value (Abalos et al. 2001). In medium supplemented with 2% glucose or higher, orcinol-sulfuric acid assay may overestimate the concentration of rhamnolipid in supernatant.

### Emulsification capacity assay

Emulsification index E24 analysis was performed according to a procedure reported previously (Cooper and Goldenberg 1987). In brief, equal amount of surfactant solution and crude oil was mixed using a vortex (IKA, Staufen, Germany) at the maximum level for 2 min and subsequently remained standstill for 24 h. The E24 index was estimated by a ratio between the emulsion volume and total content volume.

### Ribosomal RNA gene sequence-based analysis

To obtain microbial genomic DNA for PCR amplification, mixed or clonal microbial cultures were pelleted by centrifugation and the resulting pellet was resuspended in lysis solution using Genomic DNA Extraction kit (Axygen Scientific Inc., Tewksbury, MA, USA) and genomic DNA was extracted according to the manufacturer’s instruction. PCR was performed by using the microbial genomic DNA as template and 16S rDNA-specific primers 27F, 5′-AGAGTTTGATCCTGGCTCAG-3′ and 1492R, 5′-GGTTACCTTGTTACGACTT-3′) (Moreno et al. 2002). The amplification condition was after the initial denaturation at 94 °C for 5 min, 30 cycles of 94 °C for 30 s, 55 °C for 30 s, and 72 °C for 90 s, and a final extension at 72 °C for 10 min. The PCR fragment was subjected to sequencing in BGI (BGI, Shenzhen, China). The resulting sequences were compared with NCBI’s nucleotide sequences using BLAST tools (http://www.ncbi.nlm.nih.gov). Top hit sequences were downloaded and included in alignment analysis using CLUSTAL X 2.0 software (Larkin et al. 2007) and phylogenetic tree construction by MEGA 6.0 software (Tamura et al. 2013).

### TFL-RFLP analysis

For TFL-RFLP and RFLP analysis, SybrGreen labeled 27F primer was used in PCR amplification of 16S rDNA sequences. The resulting fragment was HhaI digested and separated on an 8% DNA PAGE gel. Gel image was recorded in the Gel Imaging System Tanon 5200 (Tanon Scientific Inc., Shanghai, China) in a SybrGreen fluorescence channel. For RFLP analysis, TFL-RFLP gel was imaged after ethidium bromide staining.

### qRT-PCR analysis

For analysis of transcriptional profiles of RhlA, RhlB, and RhlC genes, samples at 0, 10, 20, 35, and 60 h after growth in MS medium supplemented with 2% glucose. The transcript sequences were based on the study (Zhang et al. 2012) and sequence-specific primer pairs for RhlA, RhlB, and RhlC were RhlA_F (5′-GAAATCCTCCTGGCGCTGAT-3′) and RhlA_R (5′-ACGGTCTCGTTGAGCAGATG-3′), RhlB_F(5′-CGCATCGCTCACGAGAAGTA-3′) and RhlB_R (5′-GTCGAGTCCCTGGTTGAAGG-3′), and RhlC_F (5′-CGTGCTGGTGGTACTGTTCA-3′) and RhlC_R (5′-GTCGAGTCCCTGGTTGAAGG-3′), respectively. One-step quantitative reverse transcription PCR were performed using the Ultra SyGr One Step RT-PCR kit (Thermo Fisher Biochemicals Ltd) on a ViiA 7 Real-Time PCR System (AB Scientific, USA) machine following the manufacturer’s instruction. The RpoD was used as control for constitutively transcribe gene according to Zhang et al. (2012) and its sequence-specific primers was rpoD_F (5′-GGGCGAAGAAGGAAATGGTC-3′) and rpoD_R (5′-CAGGTGGCGTAGGTGGAGAA-3′). Transcription levels were 0-normalized.

### Purification of rhamnolipids

Extraction of rhamnolipids was performed according to the procedure previously reported (Chandankere et al. 2013). In brief, cell-free supernatant was acidified to precipitate rhamnolipids by addition of 6 N HCl to pH 2 and kept at room temperature overnight. The precipitated rhamnolipids collected by centrifugation at 4000 rpm 25 °C for 20 min. The resulting pellet was resuspended in solution of chloroform: methanol with a ratio of 2:1 and the chloroform fraction was recovered and evaporated using a distiller for chloroform recycling. This extraction of rhamnolipids was repeated for at least three times using chloroform: methanol 2:1 solution. The resulting brownish semisolid rhamnolipids were dried in oven at 70 °C overnight. The purified rhamnolipids were used for biosurfactant activity and chemical composition analyses. Rhamnolipids were resolved in distill water for surface tension and emulsification analyses and resolved in chloroform for TLC and LC–MS/MS analyses.

### Thin layer chromatographic analysis

For thin-layer chromatographic (TLC) analysis, 0.1 g purified-rhamnolipids was resolved in 1 ml chloroform. Approx. 100 μg rhamnolipids solution was loaded on to a TLC plate (Marine Biotech Co., Qingdao, China). As control, 50 μg of the standard rhamnolipids rha-C_10_-C_10_ and rha-rha-C_10_-C_10_ (Sigma/Aldrich, USA) was loaded. The plates were developed in the solution of chloroform: methanol: acetic acid at a ratio of 65:15:2 and exposed to iodine vapor for visualization of glycolipids.

### LC–MS/MS

To analyze the composition of the rhamnolipids produced by the ZS1, its purified rhamnolipids was resolved in chloroform at a concentration of 0.1 g/ml. LC–MS/MS analysis of the rhamnolipids was performed using the Water UPLC (Waters Corp., Milford, MA, USA) system equipped with the Aquity UPLC Beh-C_18_ column (1.7 μm, 2.1 × 50 mm; Waters Corp.) coupled with the AB Triple TOF 5600 plus System (AB Sciex, Framingham, USA). In LC analysis, the mobile phases 0.1% formic acid–water (A) and 0.1% formic acid-acetonitrile (B) were employed. Linear gradient programs were set as follows, 0/20, 20/95,35/95, 36/20 (min/B%); Sample injection volume was 2 μl; Column oven temperature set as 35 °C; Flow rate was 0.4 ml/min; and the UV detector was set at the wavelength of 220 nm. In mass spectrometry analysis, MS scan range was set at *m/z* 100–2000 in negative ion mode with a source voltage of −4.5 kV and source temperature at 550 °C. The pressure of Gas 1 (Air) and Gas 2 (Air) were set to 50 psi. The pressure of Curtain Gas (N2) was set to 35 psi. Maximum allowed error was set to ±5 ppm. Declustering potential (DP) was 100 V; collision energy (CE) at 10 V. For MS/MS acquisition mode, the parameters were almost the same except that the collision energy (CE) was set at 50 ± 20 V, ion release delay (IRD) at 67, ion release width (IRW) at 25. Analyst TF 1.6 and Peakview 1.2 software (AB Sciex, Framingham, USA) were used for data acquisition and data analysis, respectively.

### Analyses of surfactant activity of RL under various environmental conditions

To compare the stability of biosurfactant RL7-11 and synthetic surfactant SDS under conditions with various pH, salinities, and temperatures, aliquots of 0.12 g/l RL7-11 and 1.5 g/l SDS were subjected to various treatments according to the method described previously (Chandankere et al. 2013) prior to surface tension and emulsification index E24 analyses. For stability under various pH, aliquots of 20 ml surfactants were adjusted to pH 4.0, 6.0, 8.0, 10.0 and 12.0 using 6 M NaOH or 6 M HCl prior to surface tension and emulsification index analyses. For stability under various salinities, equal volume of twofold concentrated surfactant and NaCl solutions were mixed to have the final concentration of RL7-11 at 0.12 g/l and NaCl at 0, 4, 8, and 12% in each sample prior to surfactant activity analyses. For stability under various temperatures, aliquots were incubated at 25, 40, 60, 80, and 100 °C for 30 min prior to analyses of surfactant activity. All experiments were performed in triplicate. Commercial rhamnolipids Rha-C10-C10 and Rha-Rha-C10-C10 (RL-STD) (BALX Biotech Co. Ptd., Tianjin, China) was also used in the text at a concentration of 0.12 g/l as that of RL7-11.

### Analysis of the rate for crude oil consumption by ZS1

To estimate the consumption rate of crude oil or hexadecane by ZS1 isolate, growth curve analysis of ZS1 in shake-flask at 30 °C 180 rpm containing ME medium supplemented with 1% crude oil or hexadecane was performed. Total crude oils in the first week (due to the presence of adhesive oil residues at flask wall) of growth were determined by using replicates. Culture in flask was poured out to a fresh tube, followed by centrifugation to separate the pellet and supernatant (including floating oils). The resulting pellet was subjected to cell dry weight determination; the floating oils were poured back to the initial flask and extracted together with hexane for determination of unemulsified oils. The remaining supernatant was extracted with equal volume of hexane for determination of emulsified crude oil. A week after growth (no apparent adhesive oil residues), 10–20 ml culture was sampled for determination of cell dry weight (cell mass), floating oils and emulsified oils at various time points in triplicate. All measurements were carried out in triplicate. Total amount of crude oil is calculated as the sum of unemulsified and emulsified oils.

### Information related to strain deposition and 16S rDNA sequence submission

The sludge-derived rhamnolipid-producing *P. aeruginosa* ZS1 isolate described in this study was deposited in China General Microbiological Culture Collection Center (CGMCC) with the identity number of 13656. Its 16S rDNA sequence was submitted to GenBank at the National Center for Biotechnology Information (NCBI) with the accession number of KY437088.

## Results

### Isolation of glucose-enriched biosurfactant-producing microbes derived from oil sludge

**Fig. 1:**
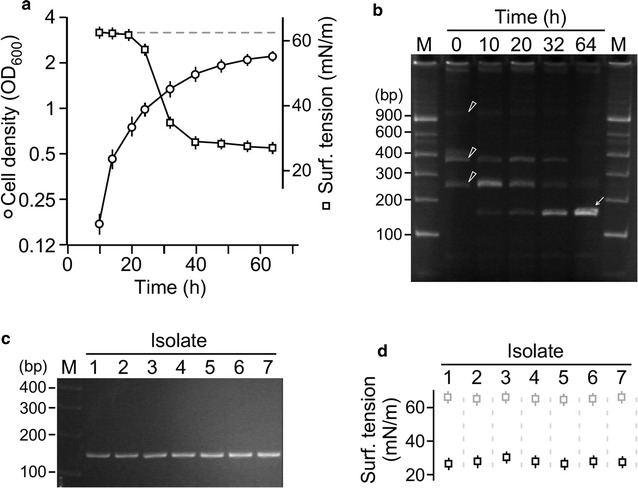
Identification of biosurfactant activity in mixed microbial cultures derived from oil sludge. **a** Surfactant activity is detected in supernatant of cultures originated from oil sludge.* X*- and* Y*-axis indicate the time and cell density (OD_600_) of the culture. *Inset* indicates the surface tension (mN/m) at various time points. The *horizontal dash line* indicates the surface tension of the fresh medium. **b** Dynamics of individual microbial populations. TFL-RFLP analysis of oil sludge-derived cultures at various time points. *Arrowhead* and *arrow* indicate the initial (at 0 h) and final (at 64 h) microbial populations, respectively. **c** Characterization of microbial isolates from cultures 64 h after growth. TFL-RFLP analysis of individual isolates, identical to those observed at 64 h in (B). **d** Biosurfactant activity produced by individual isolates. Surface tension of individual isolate cultures (*black square*) and fresh medium (*grey square*)

Petroleum sludge was collected near the Sanjiang Ferry Terminal located in Zhoushan islands, Zhejiang Province, China. Approx. 0.1 g of petroleum sludge was resuspended in the minimal salt (MS) medium (Zajic and Supplison 1972) supplemented with 2% yeast extract. The resulting culture was utilized as seed to inoculate fresh MS medium supplemented with 2% glucose as sole carbon source. Cell-free supernatant of cultures was subjected to surface tension analysis using the du Nouy ring method (Butt et al. 2003; du Noüy 1925) in triplicate (see “Materials and methods”). Surface tension of the culture supernatant at start was ~62 mN/m. After growth for 32 h, surface tension of supernatant was reduced to 37 mN/m (Fig. 1a), indicating that biosurfactants were produced in microbial culture derived from oil sludge.

To investigate the dynamics of microbial populations in cultures derived from oil sludge, we performed the rapid terminal fluorescence-labeled (TFL)-RFLP analysis to monitor the dynamics of individual microbial populations in cultures of MS medium supplemented with glucose (see “Materials and methods”). In this TFL-RFLP analysis, 16S rRNA gene sequences of the sludge-derived microbes were amplified by the standard primer pairs (fluorescence labeled 27F, 5′-AGAGTTTGATCCTGGCTCAG-3′; 1492R, 5′-GGTTACCTTGTTACGACTT-3′) (Moreno et al. 2002) and subsequently digested with restriction enzyme *Hha*I. Each microbial species possessed only one fluorescence labeled-fragment with a length depending on the position of the first *Hha*I site to the labeled primer. The analysis showed that, prior to growth in MS medium supplemented with 2% glucose, three major species with the characteristic of RFLP fluorescence fragments of 270, 380 bps, and 1.2 kb in length were present in starting culture at 0 h (Fig. 1b, see arrowhead). After growth for 64 h in MS medium supplemented with glucose, a major species with the characteristic of RFLP fluorescence fragment of 170 bps in length appeared (Fig. 1b, see arrow), while three initial species diminished.

To investigate whether the species with the characteristic of RFLP fluorescence fragment of 170 bps in length was responsible for biosurfactant production, single colonies from the culture after growth for 64 h in MS medium supplemented with glucose were obtained. Seven randomly chosen colonies grown in MS medium supplemented with 2% glucose were subjected to TFL-RFLP analysis. All seven selected colonies exhibited a characteristic of RFLP fluorescence fragment of 170 bps in length (Fig. 1c). Biosurfactant activity in supernatant of the isolate cultures were obvious (Fig. 1d). This result suggested that a biosurfactant-producing microbial species from oil sludge was enriched by glucose as sole carbon source.

### Biosurfactant-producing microbe ZS1 isolated from oil sludge belongs to the group of *P. aeruginosa*

**Fig. 2:**
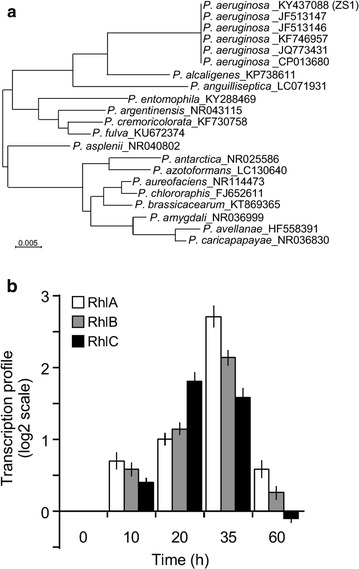
Biosurfactant-producing ZS1 isolate belongs to the group of *Pseudomonas aeruginosa*. **a** The 16S rDNA sequence-based phylogenic analysis of the ZS1 isolate. The isolate is indicated as ZS1 in *parentheses*, whose nucleotide sequence is available in Genebank (https://www.ncbi.nlm.nih.gov/genbank) with the Accession Number KF668476). **b** Transcription of rhamnolipid synthesis genes is induced in medium with glucose as sole carbon source. qRT-PCR analysis of RhlA, RhlB, and RhlC gene expression at various time-points after ZS1 growth in MS medium supplemented with glucose

To investigate the species of the biosurfactant-producing microbe isolated from oil sludge, one of the seven isolates (or ZS1 isolate) was subjected to 16S rDNA sequence analysis. For this reason, the 16S rDNA sequence was PCR amplified with sequence-specific primers (Moreno et al. 2002). The resulting DNA fragment was subjected to nucleotide sequencing analysis and followed by phylogenic analysis using CLUSTAL X 2.0 (Larkin et al. 2007) and MEGA 6.0 software (Tamura et al. 2013). Based on sequence-based analysis, ZS1 isolate appeared to fall into the group of *P. aeruginosa* (Fig. 2a). Hence, it was designated as *P. aeruginosa* ZS1.

Many members of the *P. aeruginosa* group isolated from either patients or oil sludge were known to produce rhamnolipids (Muller et al. 2012). Biosynthesis of rhamnolipids depended on three key enzymes RhlA, RhlB, and RhlC (Ochsner et al. 1994a, b; Rahim et al. 2001; Rehm et al. 1998). To test whether transcription of these genes in the ZS1 isolate was induced upon growth in medium with glucose as sole carbon source, we performed quantitative reverse transcriptase (qRT)—PCR analysis. To this end, we found that transcription of all three enzymes-encoding genes was induced with the peak at 35 h after growth in MS medium supplemented with 2% glucose (Fig. 2b). This result was consistent with the observation that biosurfactant activity appeared at 35 h after growth in medium with glucose as sole carbon source (see Fig. 1).

### Rhamnolipids produced by ZS1 isolate consists of 7 mono- and 11 di-rhamnolipids homologues

**Fig. 3:**
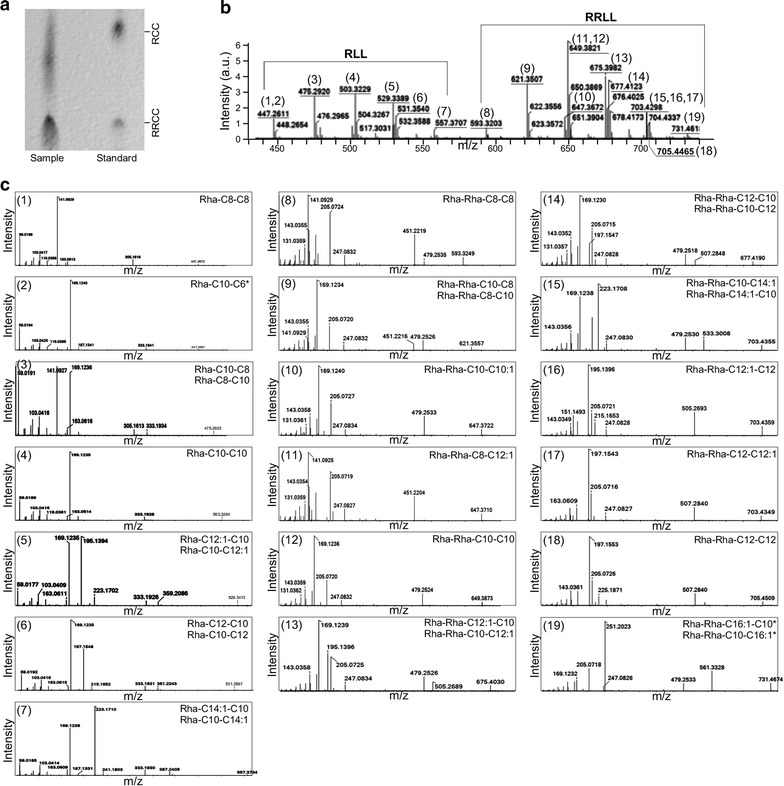
Identification of various types of rhamnolipids produced by *P. aeruginosa* ZS1. **a** Thin layer chromatographic analysis of the rhamnolipids produced by *P. aeruginosa* ZS1. Sample and standard molecules are indicated. Positions of the reference molecules Rha-C10-C10 and Rha-Rha-C10-C10 are indicated. **b** MS spectra of the rhamnolipids produced by *P. aeruginosa* ZS1.* X*- and* Y*-axis indicate m/z (mass-to-charge ratio) and intensity (arbitrary unit), respectively. *Number in parentheses* indicates various types of rhamnolipids detected in supernatant of *P. aeruginosa* ZS1. **c** MS–MS spectra of the individual peaks shown in MS spectra (**b**). Display is identical to **b**. *Top right* and *left* indicate the number of peaks shown in **b** and formulas of rhamnolipids. *Asterisk* indicates the rare type of rhamnolipids whose spectra was not reported previously

Rhamnolipids are glycolipid molecules that consist of one or two l-rhamnose sugars and one or two β-hydroxyalkanoic acids. To investigate if *P. aeruginosa* ZS1 isolate produced rhamnolipids that was responsible for the biosurfactant activity, molecules in ~4 l of cell-free supernatant containing biosurfactant activity were enriched through acidification and precipitation followed by extraction with chloroform/methanol (see “Materials and methods”). After evaporation of solvent, the resulting semi-solid materials were resolved in chloroform at a concentration of 0.1 mg/ml for TLC analysis. It was clear that the rhamnolipid mixture contained both mono- and di-rhamnolipid homologues according to standard rhamnolipid molecules of rha-C_10_-C_10_ and rha-rha-C_10_-C_10_ (Fig. 3a).

To investigate the composition of the rhamnolipid mixture produced by ZS1 isolate, supernatant extracts were subjected to LC–MS/MS analysis. To this end, the analysis showed that the mixture contained a total of 7 mono-rhamnolipid and 11 di-rhamnolipid homologues with a range of alkyl chain lengths from C_6_ to C_16_ (Fig. 3b, c). Therefore, we designated the whole mixture of rhamnolipids produced by *P. aeruginosa* ZS1 as RL7-11. Of the 7 mono-rhamnolipid homologues, 4 showed to have isoforms (rha-C_8_-C_10_/rha-C_10_-C_8_, rha-C_10_-C_12:1_/rha-C_12:1_-C_10_, rha-C_10_-C_12_/rha-C_12_-C_10_, and rha-C_10_-C_14:1_/rha-C_14:1_-C_10_), 2 had identical alkyl chain length (rha-C_8_-C_8_ and rha-C_10_-C_10_), and 1 showed no isoform detected (rha-C_10_-C_6_).

On the other hand, of the 11 di-rhamnolipid homologues, 6 showed to have isoforms (rha-rha-C_8_-C_10_/rha-rha-C_10_-C_8_, rha-rha-C_10_-C_12:1_/rha-rha-C_12:1_-C_10_, rha-rha-C_10_-C_12_/rha-rha-C_12_-C_10_, and rha-rha-C_10_-C_14:1_/rha-rha-C_14:1_-C_10_, rha-rha-C_12_-C_12:1_/rha-rha-C_12:1_-C_12_, and rha-rha-C_10_-C_16:1_/rha-rha-C_16:1_-C_10_), 3 had identical alkyl chain length (rha-rha-C_8_-C_8_, rha-rha-C_10_-C_10_, and rha-rha-C_12_-C_12_), and 2 had no isoforms detected (rha-rha-C_8_-C_12:1_ and rha-rha-C_10_-C_10:1_). We found that, of the 7 mono- and 11 di-rhamnolipid homologues, 2 rhamnolipid homologues, namely the rha-C_10_-C_6_ and rha-rha-C_16:1_-C_10_ (or rha-rha-C_10_-C_16:1_), were not previously identified. Based on the relative abundance of various homologues, common rhamnolipid homologues rha-rha-C_10_-C_10_ and rha-C_10_-C_10_ were the top 2 most abundant molecules in the RL7-11 mixture, accounting for 37.4% of the total rhamnolipids (Table 1).

**Table 1:** Composition of rhamnolipids RL7-11 produced by *P. aeruginosa* ZS1

No.	[M-H]^−^ (m/z)	RL7-11 components	Molecular formula	Relative level (%)	Rank
1	447.3	Rha-C8-C8	C22H40O9	1.47	16
2	447.3	Rha-C10-C6	C22H40O9	2.78	9
3	475.3	Rha-C10-C8/Rha-C8-C10	C24H44O9	10.11	4
4	503.3	Rha-C10-C10	C26H48O9	17.74	2
5	529.3	Rha-C10-C12:1/Rha-C12:1-C10	C28H50O9	2.36	13
6	531.4	Rha-C10-C12/Rha-C12-C10	C28H52O9	4.72	6
7	557.4	Rha-C10-C14:1/Rha-C14:1-C10	C30H54O9	0.85	18
8	593.3	Rha-Rha-C8-C8	C28H50O13	1.79	15
9	621.4	Rha-Rha-C10-C8/Rha-Rha-C8-C10	C30H54O13	11.80	3
10	647.4	Rha-Rha-C10-C10:1	C32H56O13	1.80	14
11	647.4	Rha-Rha-C8-C12:1	C32H56O13	2.71	11
12	649.4	Rha-Rha-C10-C10	C32H58O13	19.66	1
13	675.4	Rha-Rha-C12:1-C10/Rha-Rha-C10-C12:1	C34H60O13	2.37	12
14	677.4	Rha-Rha-C12-C10/Rha-Rha-C10-C12	C34H62O13	8.28	5
15	703.4	Rha-Rha-C10-C14:1/Rha-Rha-C14:1-C10	C36H64O13	3.62	8
16.1	703.4	Rha-Rha-C12:1-C12	C36H64O13	0.94	17
16.2	703.4	Rha-Rha-C12-C12:1	C36H64O13	3.77	7
17	705.5	Rha-Rha-C12-C12	C36H66O13	2.72	10
18	731.5	Rha-Rha-C10-C16:1/Rha-Rha-C16:1-C10	C38H68O13	0.50	19

### Rhamnolipid yield in ZS1 isolate is affected by concentration of supplemented carbon sources

**Fig. 4:**
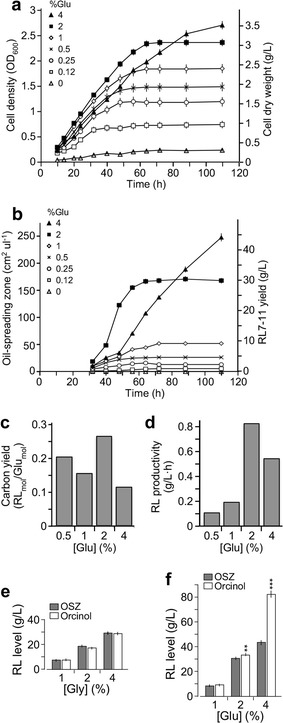
Rhamnolipid yield by ZS1 isolate under various growth conditions. **a** Growth curve analysis of ZS1 cultures with various amounts of glucose supplements.* X*- and* Y*-axis indicate time in hour (h) and cell concentration in optical density (*left*) or cell dry weight (*right*), respectively. **b** Change of surfactant activities during cell growth.* X*- and* Y*-axis indicate time (h) and oil-spreading zone (cm^2^/μl, *left*) or RL7-11 concentration (g/l, *right*). **c** Carbon yield or ratio between product RL7-11 and carbon source glucose in ZS1 cultures with various glucose supplements.* X*- and* Y*-axis indicate supplement glucose concentration and carbon yield in RL_mol_/Glu_mol_, respectively. **d** RL7-11 productivity in ZS1 cultures with various glucose supplements.* X*- and* Y*-axis indicate supplemented glucose concentration and RL7-11 productivity in g/l h, respectively. **e** Quantification of rhamnolipids by oil-spreading assay (OSZ) or orcinol method (Orcinol) in cultures with various glycerol supplement. No significantly differences between the two methods is observed. **f** Quantification of rhamnolipids by oil-spreading assay (OSZ) or orcinol method (Orcinol) in cultures with various glucose supplement. Significant difference between the two methods is apparent. *Asterisk* indicates the significant difference between the results by OSZ and Orcinol

To investigate whether rhamnolipid yield in ZS1 isolate was altered by change of glucose supplement concentration in MS medium, we performed analyses of cell biomass and biosurfactant activity by ZS1 in MS medium supplemented with various concentrations of glucose (see “Materials and methods”). We found that the log-phase growth rate of the cultures was positively correlated with glucose concentrations from 0.12 to 2%, but not 4% (Fig. 4a). On the other hand, the cell density at the onset of stationary-phase was correlated with the glucose concentration from 0.12% up to 4%. This result indicted that increase of glucose concentrations enhanced the maximum cell density of the ZS1 culture.

Biosurfactant activity in supernatant of cultures with various concentrations of glucose supplements at different time points during growth was determined using oil-spreading assay. We found that the surfactant activity was detected at the early log-phase and soon reached maximal level at the onset of stationary phase (Fig. 4b). Oil-spreading analyses indicated that areas of oil-spreading zone in supernatant of ZS1 cultures containing 0.5, 1, 2 and 4% glucose supplements were 25, 50, 170, and 250 cm^2^/μl, respectively. Based on a standard curve (see “Materials and methods”), the sizes of oil-spreading zone could be approximated to concentrations of rhamnolipids RL7-11 at 4.7, 9.3, 30, and 44 g/l.

We found that ZS1 isolate in MS medium supplemented with 2% glucose showed the maximum level of glucose usage or carbon yield of 26.8% mol rhamnolipid/mol glucose compared to that of cultures with 0.5, 1, and 4% glucose supplements (Fig. 4c). Additionally, ZS1 culture with 2% glucose supplement exhibited the maximum productivity of 827 mg/l h compared to that of cultures with 0.5, 1, and 4% glucose supplements (Fig. 4d).

To investigate whether ZS1 isolate produced rhamnolipids using glycerol as sole carbon source, we analyzed the growth of ZS1 in MS medium supplemented with 1, 2, and 4% glycerol. Maximum yield of ZS1 cultures supplemented with 1, 2, and 4% glycerol was 7.5, 18.5 and 30 g/l (respectively) based on both oil-spreading zone (Morikawa et al. 2000) and orcinol sulfuric acid methods (Abalos et al. 2001) (Fig. 4e). Given glycerol as by-product of biodiesel production, using crude glycerol by-product could reduce the cost of rhamnolipid production.

We determined rhamnolipid content in cultures supplemented with 1, 2 and 4% glucose using both oil-spreading zone method and orcinol sulfuric acid method. The level of rhamnolipid determined by orcinol sulfuric method was found to be much higher than that by oil-spreading zone when concentration of glucose supplements was 2% or higher (p < 0.01) (Fig. 4f). This result suggested that glucose residue remained in culture medium might contribute to the overestimation of rhamnolipid yield. Hence, rhamnolipid levels in glucose-containing media were estimated using oil-spreading method throughout this study.

### Rhamnolipid mixture RL7-11 is highly effective compared to the chemically synthesized SDS

**Fig. 5:**
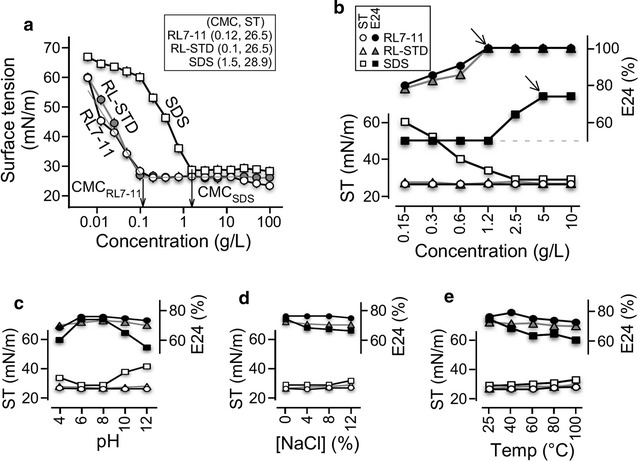
Comparative analysis of surfactant activity between RL7-11, RL-STD, and SDS. **a** Analysis of CMC-RL7-11, CMC-RL-STD, and CMC-SDS.* X*- and* Y*-axis indicate surfactant concentration in g/l and surface tension in mN/m, respectively. **b** Analysis of surface tension and emulsification capacity E24 index against crude oil. *Solid* and *open marks* indicate surface tension in mN/m and E24 index in percent. *Circle*, *triangle*, and *square* indicate RL7-11, RL-STD, and SDS, respectively. Analysis of surfactant activity under various pH, salinity, and temperature conditions is shown in **c**, **d** and **e**, respectively. The display is identical to **b**

Critical micelle concentration (CMC) defined the minimum concentration required for micelle formation, indicating the efficacy of surfactants. To compare the efficacy between rhamnolipid mixture RL7-11 and chemically synthetic surfactant SDS, we first determined CMC for RL7-11 and SDS. To this end, 10% (w/v) initial solutions of RL7-11 and SDS were subjected to twofold serial dilutions and subsequently measurement of surface tension using the du Nouy ring method (du Noüy 1925). The analyses indicated that rhamnolipids RL7-11 reduced surface tension to the minimum level of 26.4 mN/m at a concentration of 0.12 g/l, or CMC (Fig. 5a). On the other hand, the synthetic surfactant SDS showed a CMC of 1.5 g/l that reduced surface tension to the minimum level of 28.9 mN/m. We noted that reference rhamnolipids containing rha-C10-C10 and rha-rha-C10-C10 (or RL-STD) whose CMC was 0.1 g/l, a bit lower than that of RL7-11. This result indicated that rhamnolipids RL7-11 and RL-STD exhibited higher capability in terms of reducing surface tension (26.4 mN/m versus 28.9 mN/m) at lower concentration of compounds (0.12 or 0.1 g/l versus 1.5 g/l) compared to the synthetic surfactant SDS.

We subsequently determined emulsification E24 indexes of surfactants in various concentrations to compare the emulsification capacity against crude oil between RL7-11, RL-STD, and SDS. Our analysis indicated that RL7-11 and RL-STD reached the maximum E24 index of 100% at the minimum concentration of 1.2 g/l, whereas SDS reached the maximum E24 index of 74% at the minimum concentration of 5 g/l (Fig. 5b). This result indicated that RL7-11 and RL-STD exhibited higher emulsification capacity against crude oil than did SDS.

We next compared the stability of surfactant activity such as surface tension reduction and emulsion formation and maintenance between RL7-11, RL-STD, and SDS under various environmental conditions. To ensure the adequate comparison, concentrations of RL7-11, RL-TSD, and SDS at 0.12, 0.12, and 5 g/l (respectively) were employed in the analysis. RL7-11 and RL-STD at the concentration of 0.12 g/l under optimal conditions (pH7, 25 °C, without salt) reduced surface tension to 26.5 mN/m and exhibited emulsification E24 index of 76% against crude oil, while SDS at 5 g/l reduced surface tension to 29 mN/m and displayed emulsification E24 index of 74%.

It was clear that RL7-11 displayed a consistent activity in reducing surface tension to the minimum level and maintaining emulsion under wide ranges of pH (pH 4–12), salinity (0–12% NaCl), and temperature (25–100 °C) (Fig. 5c–e). In contrast, SDS showed activity within a relatively narrow range of pH (6–8), salinity (0–4% NaCl for emulsification; 0–8% NaCl for surface tension reduction), and temperature (25–60 °C for emulsification, 25–80 °C for surface tension reduction). Hence, we concluded that the RL7-11 was a better surfactant for applications in environmental remediation. It appeared that RL-STD showed a better performance than that of SDS under various pH, salinity, and temperature conditions, but not as stable as RL7-11.

### Cell mass increase of ZS1 is accompanied with emulsification and consumption of crude oil

**Fig. 6:**
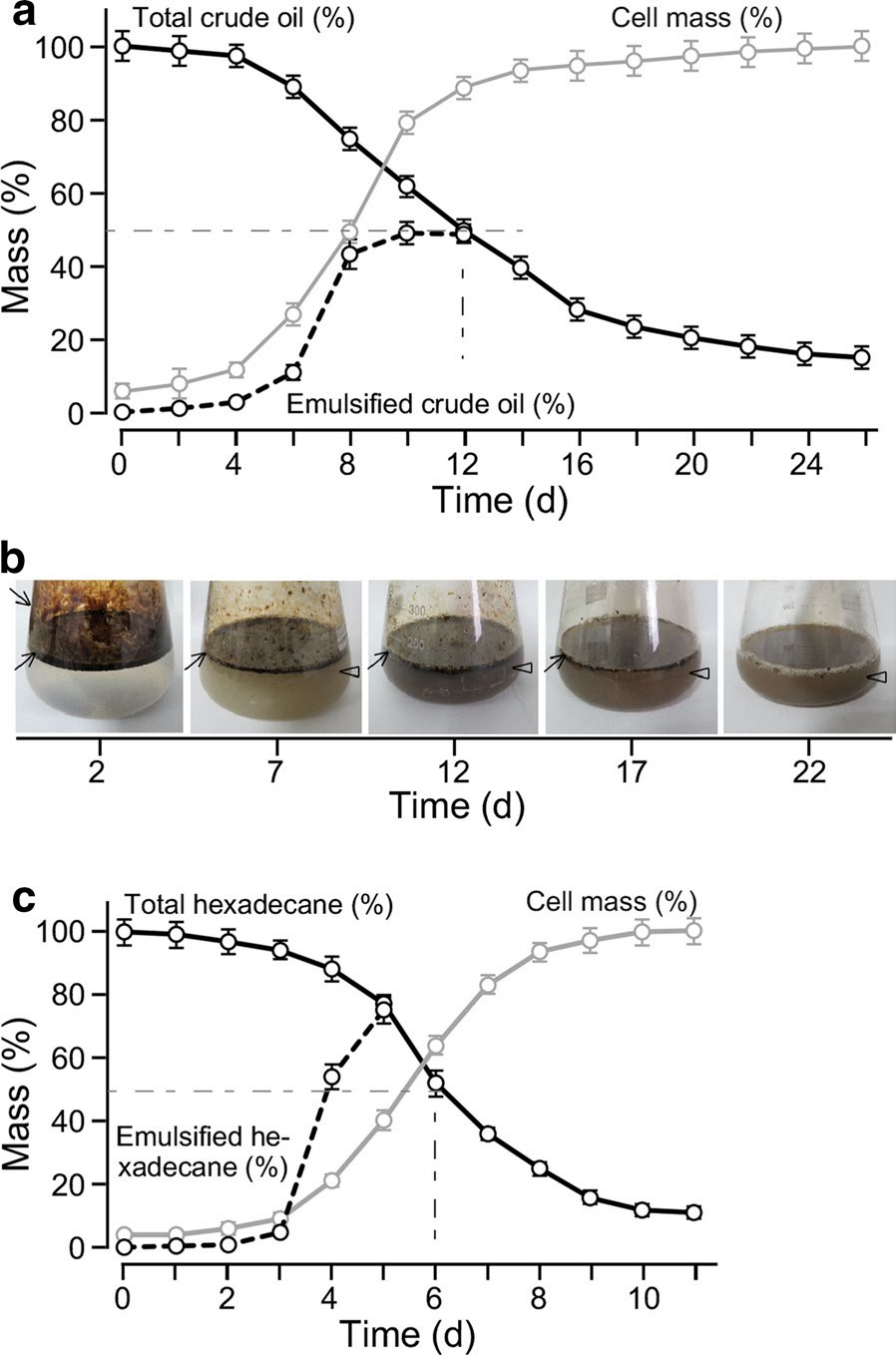
ZS1 growth in medium with crude oil as sole carbon source. **a** Growth curve analysis of ZS1 isolate in MS medium supplemented with 1% crude oil. X- and Y-axis indicate time (days) and percent of mass (%) of crude oil or cell biomass. Maximum level (or 100%) of crude oil and cell biomass are 10 and 6.5 g/l, respectively. *Dash-line* indicates 50% crude oil remained after growth for 12 day. **b** Degradation of crude oil by ZS1 isolate. Images show crude oil in shake-flask containing ZS1 culture at various time points during growth. *Arrowhead* and *arrow* indicate the unemulsified and emulsified crude oils, respectively. **c** Growth curve analysis of ZS1 isolate in MS medium supplemented with 1% hexadecane. The display is identical to **a**

To investigate whether ZS1 isolate was capable of utilizing crude oil for growth, which was essential for its potential application of in situ bioremediation, we performed growth analysis of ZS1 isolate in MS medium supplemented with 1% crude oil as sole carbon source (Fig. 6a). Level of total (unemulsified and emulsified) crude oil and cell biomass was determined after separation by centrifugation using gravimetric methodologies (see “Materials and methods”). Upon inoculation of ZS1 in fresh MS medium supplemented with crude oil, we found that crude oil was either adhered to sidewall of the shake-flack or floated on top of the culture (Fig. 6b, see arrow). Oil emulsion became visible 6 h after growth, which coincided with the cell biomass increase (Fig. 6a, b, see arrowhead). Twelve days after growth, 50% of total crude oil was consumed by the ZS1 cells, exhibiting a degradation rate of 417 mg/l days.

We subsequently investigated the consumption of hexadecane as petroleum product by ZS1 isolate. Based on the same approach, we found that 50% of total hexadecane was consumed in 5.5 days (Fig. 6c). This result indicated that ZS1 utilized refined petroleum product more effectively than crude petroleum oil.

## Discussion

Biosurfactants provide a promising alternative to chemically synthesized surfactants because of their low toxicity, high biodegradability, and ecological safety (Sponza and Gök 2010). Rhamnolipids are thought to be the next-generation surfactants for its high efficacy and stability over various environmental conditions (Henkel et al. 2012; Sponza and Gök 2010). In this study, we show the isolation of a rhamnolipid-producing *P. aeruginosa* ZS1 from petroleum sludge in Zhoushan islands, China, where a national strategic petroleum reservoir is located. The ability of petroleum consumption by ZS1 makes it suitable for cleanup of petroleum pollutants in Zhoushan.

Many methods employed in screening for biosurfactant-producing microbes from petroleum pollutants in marine and terrestrial environments are often involved in firstly the separation and isolation of individual microbial colonies on plates with indicator for surfactant activities (Walter et al. 2010). Because biosurfactants may not be constitutively produced in microbes, they will either be missed during the screening or multiply the workload for screening colonies on plates under different growth conditions. To circumvent this issue, we screen biosurfactant activities in mixed cultures. Changes of microbial populations in mixed culture were monitored by a rapid real-time TFL-RFLP method.

Several environmental isolates of *P. aeruginosa* have shown to produce rhamnolipids (Abalos et al. 2001; Abdel-Mawgoud et al. 2009; Ma et al. 2016). Some isolates favor lipids instead of glucose as carbon sources (Ma et al. 2016). Additionally, ratios between carbon and nitrogen sources (C/N) affect rhamnolipid yields (Ma et al. 2016). In this analysis, we show that ZS1 produces rhamnolipids up to 30 and 44 g/l at the C/N ratio of 12:1 and 24:1 (respectively) in medium with glucose as sole carbon sources. Furthermore, ZS1 is capable of consuming crude oil, which make it ideal for bioremediation. These observations are consistent with the notion that nutrient preferences are isolate-dependent (Wu et al. 2008).

Up to now, the highest rhamnolipid yields are reported to be ranged from 70 to 120 g/l by the chemically mutagenized strains DSM 7107 and DSM 7108 derived from an environmental isolate cultivating in bioreactors (Giani et al. 1997). On the other hand, the highest rhamnolipid yields from the naturally occurring isolates growing in a shaking flask are found to be up to 9.5 and 25.9 g/l (Abalos et al. 2001; Ma et al. 2016). In this study, we show that ZS1 isolate give rise to maximum yields of 30 and 44 g/l after 64 and 110 h growth in MS medium supplemented with 2 and 4% glucose, respectively. Among the naturally occurring isolates growing in shake-flasks reported so far, the ZS1 gives the highest rhamnolipid yield. This is probably attributed to the screening in mixed population of sludge-derived microbes instead of individually isolated colonies, because microbes responsible for the strongest surfactant activities in the mixed population are readily monitored. The rapid TFL-RFLP assay on dynamics of microbial populations facilitates the identification of the surfactant-producing microbes.

Rhamnolipids are often referred as a mixture of monorhamnolipid rha-C_10_-C_10_ and dirhamnolipid rha-rha-C_10_-C_10_ (Syldatk and Wagner 1987). Many rhamnolipids produced in *P. aeruginosa* are found to contain multiple rhamnolipid homologues besides the most common forms (Abalos et al. 2001; Abdel-Mawgoud et al. 2009; Ma et al. 2016). In this study, we show that the ZS1 isolate produces 7 homologues of monorhamnolipids and 11 homologues of dirhamnolipids with the most dominant homologues of rha-rha-C_10_-C_10_ and rha-C_10_-C_10_ (see Table 1), consistent with the notion that the common forms of rhamnolipid homologue are most dominant in rhamnolipid-producing microbes (Schenk et al. 1995; Zhang and Miller 1994). We show that the CMC of RL7-11 is a bit higher than that of RL-STD containing mainly the rha-C10-C10 and rha-rha-C10-C10 (CMC 0.12 g/l versus 0.1 g/l). This could be caused by the different purities of the chemical. Notably, RL mixture containing multiple homologues appear to improve the stability under various environmental stress conditions tested (see Fig. 5).

Recently, a *P. aeruginosa* NCIM 5514 isolate has been suggested as a suitable strain for bioremediation of hydrocarbon pollutants in petrochemical industry (Varjani and Upasani 2016). In this study, we show that ZS1 isolate is capable of consuming 60% crude oil at 30 °C in 14 days. In comparison, the NCIM 5514 isolate is able to degrade 60% crude oil at 37 °C in 60 days. The difference may be attributed to the high yield of rhamnolipids produced by ZS1 isolate. Hence, we concluded that ZS1 isolate is ideal for bioremediation of petroleum pollutants, especially in the coastal areas of Zhoushan.

In conclusion, our analyses show that *P. aeruginosa* ZS1 isolated from petroleum sludge produces biosurfactant rhamnolipids in minimal salt medium supplemented glucose, glycerol or crude oil as sole carbon source. Consumption of crude oil by ZS1 isolate is facilitated with its rhamnolipid secretion. Hence, we propose that the ZS1 isolate is suitable for bioremediation of petroleum pollutants, especially in the coast areas of Zhoushan islands.

## Abbreviations

GC–MS/MS: gas chromatography coupled with tandem mass spectrometry
Rha: rhamnolipid
ST: surface tension
TFL-RFLP: terminal fluorescence labeled-restriction fragment length polymorphism
TLC: thin layer chromatography
